# Morpho-Anatomical, Physiological, and Mineral Composition Responses Induced by a Vegetal-Based Biostimulant at Three Rates of Foliar Application in Greenhouse Lettuce

**DOI:** 10.3390/plants11152030

**Published:** 2022-08-04

**Authors:** Petronia Carillo, Veronica De Micco, Michele Ciriello, Luigi Formisano, Christophe El-Nakhel, Maria Giordano, Giuseppe Colla, Youssef Rouphael

**Affiliations:** 1 Department of Environmental, Biological and Pharmaceutical Sciences and Technologies, University of Campania “Luigi Vanvitelli”, Via Vivaldi 43, 81100 Caserta, Italy; 2 Department of Agricultural Sciences, University of Naples Federico II, 80055 Portici, Italy; 3 Department of Agriculture and Forest Sciences, University of Tuscia, 01100 Viterbo, Italy

**Keywords:** protein hydrolysate, *Lactuca sativa* L., anatomical traits, leaf gas exchange, inorganic ions, polyphenols, SPAD

## Abstract

A promising strategy for sustainably increasing the quality and yield of horticultural products is the use of natural plant biostimulants. In this work, through a greenhouse experiment, we evaluated the effect of a legume-derived biostimulant at three dose treatments (0.0 control, 2.5 mL L^−1^, and 5.0 mL L^−1^) on the yield performance, nutrients traits, leaf anatomical traits, gas exchanges, and carbon photosynthetic assimilation of greenhouse lettuce. The lettuce plants were foliar sprayed every 7 days for 5 weeks. The application of plant biostimulant, at both lower and higher dosages, increased the nutrient use efficiency, root dry weight, and leaf area. However, it is noteworthy that the 5.0 mL L^−1^ dose enhanced photosynthetic activity in the early phase of growth (15 DAT), thus supplying carbon skeletons useful for increasing the number of leaves and their efficiency (higher SPAD), and for boosting nutrient uptake (P, S, and K) and transport to leaves, while the 2.5 mL L^−1^ dose exerted specific effects on roots, increasing their dimension and enabling them to better use nitrate and Ca. A higher dose of biostimulant application might find its way in shorter growing cycle, thus presenting new horizons for new lines of research in baby leaves production.

## 1. Introduction

The exponential growth of the population and an increase in the demand for food have boosted traditional agriculture towards an excessive use of synthetic chemicals. Considering that fertilizers—and, in particular, nitrogen (N)—represent the highest input cost for many crops since their production is energy intensive [1], high-input farming systems are no longer sustainable, both from an environmental and energy cost perspective. Therefore, it is necessary to find a strategy to improve the nutrients use efficiency (NUE) while maintaining the productivity and quality of crops. In this context, the use of biostimulants, consisting of substances and/or microorganisms capable of enhancing crop NUE, mitigating the negative effects of abiotic stress and increasing the quality of crops is of particular interest. Plant biostimulants (BP) are neither fertilizers nor pesticides and do not have a direct effect on parasites and pathogens [2]. BP are able to improve the nutritional status of plants due to synergistic and/or additive effects dealing with an enhancement of NUE [3,4,5,6,7,8], higher soil organic matter mineralization by microorganisms [9,10,11,12,13], improved nutrients’ mobility and solubility [2,14,15,16,17,18], changes of root architecture and spatial configuration in the soil to better access to nutrients and water [19,20,21,22,23,24], up-regulation of transporters and enzymes to increase nutrients uptake and assimilation [25,26,27,28,29,30]. Among PB, plant-derived protein hydrolysate (PH), mixtures of polypeptides, oligopeptides, and amino acids, produced through partial hydrolysis of agri-food residues, can be an eco-friendly strategy to ensure development of the agricultural sector [31,32,33]. They are mainly produced through the thermal and enzymatic hydrolysis of plant residual biomasses [2,25,32]. In addition to amino acids and peptides, PH principal components are trace amounts of mineral elements, carbohydrates, phenols, phytohormones, and other metabolites able to improve germination, yield and nutritional quality of plant crops [25,32,34]. When they are applied to soil by supplying available C and N to microorganisms in the rhizosphere, they enhance soil microbial activity and soil respiration [35]. Moreover, they can function as a source of aggregate formation and chelates among micronutrients (e.g., Fe, Mn, Cu, and Zn), increasing minerals availability, root nutrient uptake, and NUE [2,35,36]. Bioactive peptides contained in plant-derived PH may act as hormone-like regulators (similar to auxin and gibberellins), enhancing shoot and root growth, modifying root architecture (e.g., number, length, surface, and density of lateral roots), and increasing crop nutrient uptake capacity (in particular of N and Fe) and yields [28,32,37,38]. An alfalfa (*Medicago sativa* L.) plant-derived PH, containing indole-3-acetic acid (IAA) and triacontanol (a saturated straight chain primary alcohol present in alfalfa wax), induced the accumulation of K, proline, and flavonoids, thus increasing plant salt stress tolerance in corn under salinity conditions [27]. Similarly, a legume seeds-derived PH was able to enhance the accumulation of compatible compounds, glucosinolates, sterols, and terpenes in lettuce (*Lactuca sativa* L.) plants under salinity conditions, thus mitigating oxidative stress and improving root dry weight, dry biomass, and fresh yield [38]. The same biostimulant was able to increase salicylates while decreasing cytokinins in tomato plants under drought, enacting an oxidative stress mitigation mediated by carotenoids, hydroxycinnamic amides, and prenyl quinones [39]. PH may improve N use efficiency by acting on the regulation of key enzymes of N metabolism (e.g., nitrate reductase and glutamine synthetase), and of tricarboxylic acid (TCA) cycle, which works as a central hub for the interacting pathways of C and N metabolism [35,40,41]. Moreover, it can induce a prodromal metabolic reshaping to improve plant crops photosynthetic performance, yield and quality in basil, perennial wall rocket and spinach [33,42,43].

The above-mentioned studies clarified the effects and efficiency of plant-derived PH depending on species and/or cultivars, cultivation, growing seasons, and mode of applications. However, few studies have investigated the dose application effects of plants biostimulants [44,45] and in particular of plant-derived PH [46]. In this view, we investigated the effect of a legume-derived biostimulant on the yield performance, nutrients traits, leaf anatomical traits, gas exchanges, and carbon photosynthetic assimilation of greenhouse lettuce by comparing three PH dose treatments: 0.0 (control), 2.5 mL L^−1^ (recommended dose), and 5.0 mL L^−1^ as a potential beneficial incremented dose.

## 2. Results

### 2.1. Yield and Biometric Parameters

**Figure 1:**
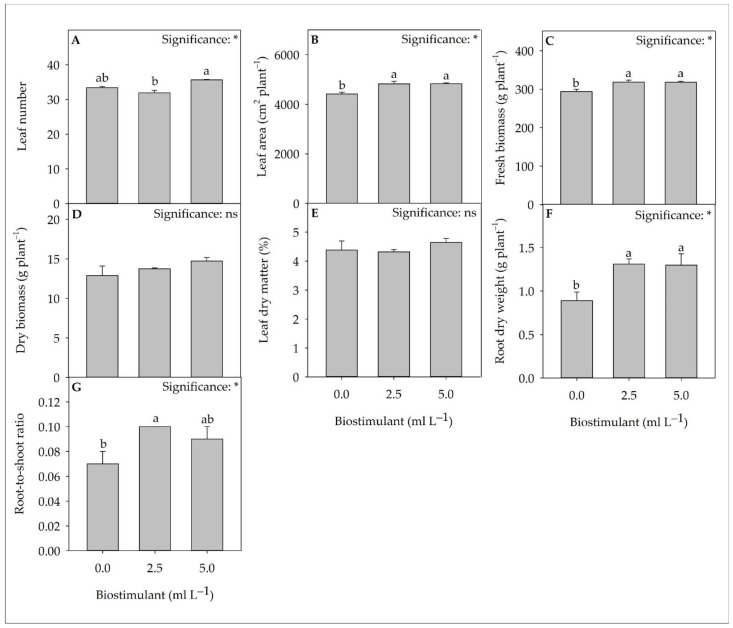
The analysis of variance and mean comparisons for leaf number (**A**), leaf area (**B**), fresh biomass (**C**), dry biomass (**D**), leaf dry matter (**E**), root dry weight (**F**), and root-to-shoot ratio (**G**) of greenhouse lettuce cultivated under three biostimulant doses. ns and * indicate non-significant or significant difference at *p* < 0.05. Different letters indicate statistically different groups according to the Tukey HSD post-hoc test (*p* = 0.05). Data are mean values ± standard error, *n* = 3.

The yield and biometric parameters of lettuce grown with two different doses of biostimulant (2.5- and 5.0-mL L^−1^) compared to control (no biostimulant) are reported in Figure 1. Save for dry biomass and leaf dry matter, all parameters were affected by the biostimulant treatment. Compared to control, biostimulant use increased, on average, leaf area, fresh biomass, and root dry weight by 9.3, 8.3, and 46.6%, respectively. The 5.0 mL L^−1^ dose, compared to the 2.5 mL L^−1^ one, increased the number of leaves by 11.7%. In contrast, no significant differences were observed in the root-to-shoot ratio between the two doses of biostimulant. On the contrary, the 2.5 mL dose increased this parameter by 42.8%, compared to the control.

### 2.2. Leaf Gas Exchange Parameters

Table 1 shows the physiological parameters analyzed (ACO_2_, E, and WUEi) at three different periods of the growth cycle, 15, 23, and 37 days after transplantation (DAT), respectively. Regardless of the DAT, the parameters E and WUEi were not influenced by the biostimulant dose, unlike ACO_2_. For the latter parameter, at 15 DAT, a 69.8% increase was observed with the use of the 5.0 mL L^−1^ dose compared to the control, while at 23 DAT, regardless of dose, the use of the biostimulant increased, on average, ACO_2_ by 42.8% compared to the control. For the SPAD index, biostimulant treatment at 5.0 mL L^−1^ dose also increased significantly compared to control.

### 2.3. Macroelements

**Figure 2:**
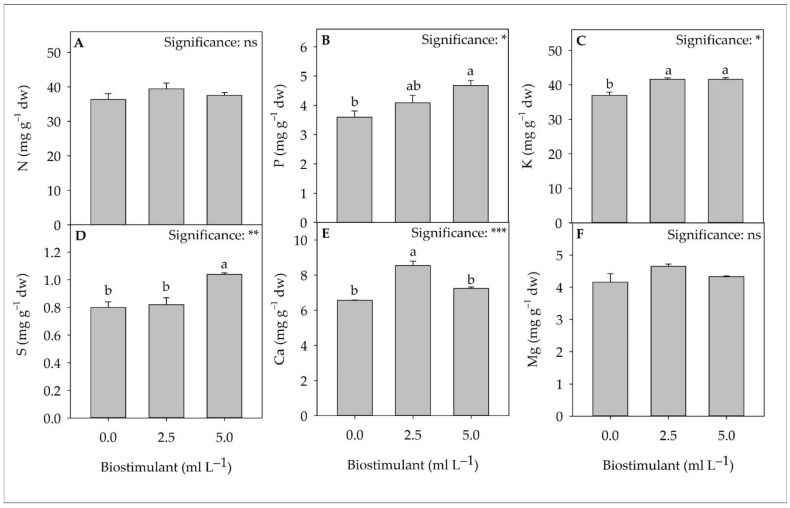
The analysis of variance and mean comparisons for nitrogen (**A**), phosphorus (**B**), potassium (**C**), sulfur (**D**), calcium (**E**), and magnesium (**F**) of greenhouse lettuce cultivated under three biostimulant doses. ns, *, **, and *** indicate non-significant or significant difference at *p* < 0.05, *p* < 0.01, and *p* < 0.001, respectively. Different letters indicate statistically different groups according to the Tukey HSD post-hoc test (*p* = 0.05). Data are mean values ± standard error, *n* = 3.

Save for N and Mg, all macronutrients quantified by ion chromatography showed significant differences between treatments. Compared to the control, the use of biostimulant at a dose of 5.0 mL L^−1^ increased the concentration of P by 30.0%. Lettuce plants treated with the same dose of biostimulant (5.0 mL L^−1^) recorded the highest S values (1.04 mg g^−1^ dw), while for Ca, the highest values (8.54 mg g^−1^ dw) were obtained with the 2.5 mL L^−1^ dose. For K, the most abundant macroelement (Figure 2), the use of the biostimulant (regardless of dose) increased its concentration compared to the control (+12.5%).

### 2.4. Leaves Quality

CIELab colorimetric parameters, the nitrate content, the total phenols, and the hydrophilic and ABTS antioxidant activities are shown in Table 2. For the colorimetric parameters of CIELab, significant differences were recorded only for L*, with the highest values (47.54) obtained in the control plants. Significant differences were also observed after biostimulant treatment for nitrate concentration. Specifically, compared to the control, using the 2.5 mL L^−1^ dose increased the nitrate value in the leaves (+14.9%), while using the 5.0 mL L^−1^ dose did not result in significant differences.

### 2.5. Leaves Morpho-Anatomical Traits

**Figure 3:**
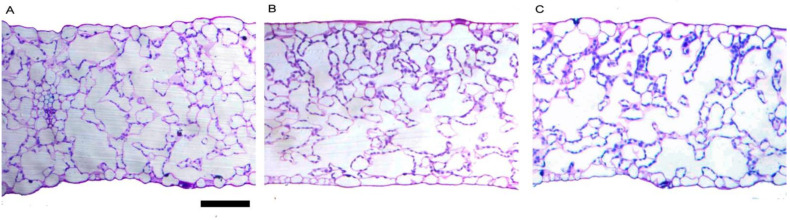
Light microscopy views of cross sections of leaf lamina of lettuce treated with the different doses of biostimulants: (**A**), control; (**B**), 2.5 mL L^−1^; (**C**), 5.0 mL L^−1^. Images are all at the same magnification. Bar = 100 µm.

Light microscopy observations of the cross sections of the leaf lamina of lettuce leaves treated with different doses of biostimulants indicated that there were no modifications in the tissue organization (Figure 3). Table 3 shows the main quantitative anatomical traits. Save for the frequency of stomatal in the adaxial lamina, all parameters were not significantly affected by biostimulant dose treatments. Compared to the control, the 5.0 mL L^−1^ biostimulant dose increased stomatal frequency in the adaxial lamina by 22.8%.

### 2.6. Principal Component Analysis (PCA)

**Figure 4:**
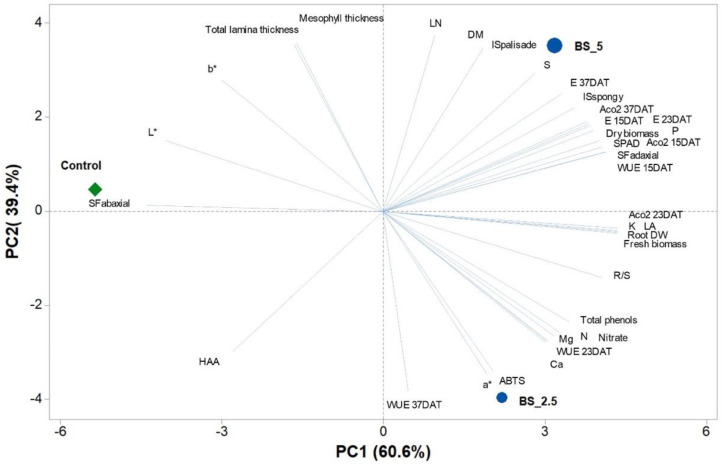
The principal component loading plot and scores of principal component analysis (PCA) of biometric traits, leaf colorimetric parameters, mineral nutrients, gas exchanges, bioactive compounds, and antioxidant activity in lettuce under three PH dose treatments (0.0, 2.5 and, 5.0 mL L^−1^).

A principal component analysis was performed on all data in relation to the three biostimulant doses (0, 2.5, and 5.0 mL L^−1^), as reported in Figure 4. The variables in the first two principal components (PCs) were highly correlated, with eigen values greater than 1, thus explaining for 100% of the total variance, with PC1 and PC2 accounting for 60.6%, 39.4%, respectively. PC1 was positively correlated to K, ACO_2_ 23 DAT, root dw, fresh biomass, leaf area (LA), stomatal frequency on adaxial lamina (SFadaxial), WUE 15 DAT, SPAD, root-to-shoot ratio (R/S), ACO_2_ 15 DAT, P, and dry biomass. While it was negatively correlated only to stomatal frequency on abaxial lamina (PC2 was positively correlated to LN, mesophyll thickness, total lamina thickness, dry matter percentage, the percentage of intercellular spaces in palisade parenchyma (ISpalisade), S and b*; while PC2 was negatively correlated to WUE 37 DAT, a*, ABTS, HAA, and Ca. The three lettuce treatments were clearly separated in respect to PC1 and PC2. In fact, control treatment (no biostimulant) was present in the negative side of PC1 in the upper left quadrant close to the x axis and clustered with SFabaxial and L*. While 2.5- and 5.0-mL L^−1^ treatments were distributed in the positive side of PC1 in the lower right quadrant and upper right quadrant, respectively (Figure 1). Interestingly, the 2.5 mL L^−1^ treatment showed the highest values of a*, ABTS, Ca and WUE 37 DAT, while 5.0 mL L^−1^ treatment showed the highest intercellular spaces in palisade and spongy parenchyma, S, leaf number, and dry matter (Figure 4).

### 2.7. Heat Map Analysis

**Figure 5:**
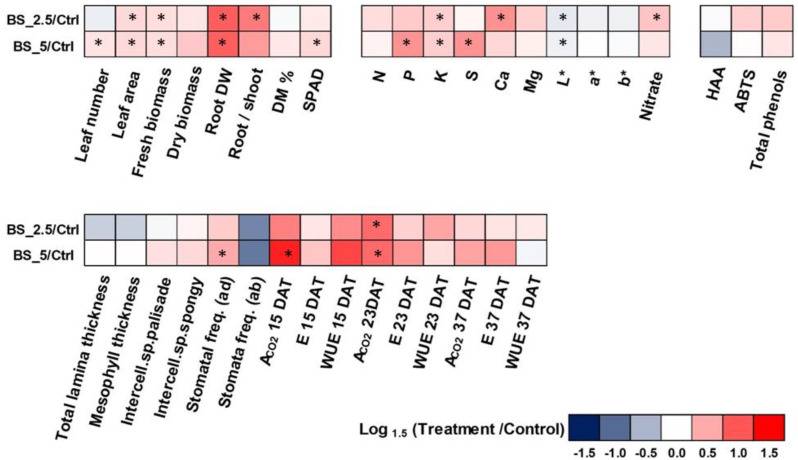
A heat map analysis summarizing lettuce plant responses to PH biostimulant 0.0, 2.5 and, 5.0 mL L^−1^ dose. The heat map results were calculated as Logarithm base 1.5 (Log_1.5_) of Treatment/Control values and visualized using a false-color scale, with red indicating an increase and blue a decrease of values. No differences were visualized by white squares. * indicates significant difference at *p* < 0.05.

An Excel-based heat map was used to sum up the effects in terms of dependence on the different PH dose provided on lettuce performance (Figure 5). It is interesting to note that the 2.5 mL L^−1^ PH dose application significantly increased the root to shoot ratio, Ca and nitrate, whereas the 5.0 mL L^−1^ treatment significantly increased the leaf number, SPAD, P, S, stomatal frequency on adaxial lamina, and ACO_2_ at 15 DAT. However, both the PH doses significantly increased leaf area, fresh biomass, root dw, K, L*, and ACO_2_ at 23 DAT.

## 3. Discussion

Over the last decade, the use of biostimulants turned out to be a promising ecological strategy to increase the quality and yield of plant produce by environmentally friendly cultivation methods. In particular, several experiments were performed to study the effect and efficiency of PBs depending on species and/or cultivars, cultivation, growing seasons, and modes of application, as detailed in the introduction, but only a few of them evaluated the effect of PBs [44,45]—specifically, plant-derived PH [46]—and dose treatments on plant performance. Therefore, we investigated the effect of three dose treatments (0 control, 2.5 mL L^−1^, and 5 mL L^−1^) of the commercial legume-derived biostimulant PH (Trainer^®^) on yield, nutrients content, leaf anatomical traits, gas exchanges, and carbon photosynthetic assimilation of greenhouse lettuce.

In our study, the application of the PH increased the leaf area, fresh yield, and root dw, independently of the dose application. The beneficial effects on plant growth and yield of PH in hydroponic systems previously seen in basil (*Ocimum basilicum* L.) [33], celery (*Apium graveolens* L.) [47], lettuce [48], and rocket (*Eruca sativa* M.) [43,49] were attributed to the bioactive amino acids and peptides present in the commercial formulations [48]. The increase of root biomass and/or modification of root architecture and the consequent increase in crop growth and yield were specifically ascribed to the hormone-like activity of the peptides contained in PH [32,37,50,51]. In particular, Matsumiya and Kubo [52] demonstrated that peptides derived from soybean hydrolysis promoted the growth of root hairs and consequently of the entire plants in eggplant (*Solanum melongena* L.), Indian mustard (*Brassica juncea*), and tomato (*Solanum lycopersicum* L.). Moreover, a sunflower defatted seed meal hydrolysate showed an auxin-like stimulation of root length able to ameliorate transplanting, crop growth, and yield in garden cress (*Lepidium sativum* L.) and lettuce [53], whereas the increase in fresh yield was attributed to the ability of amino acids, contained in the culture media supplemented with PH, and easily taken up by roots to function as signaling molecules able to promote nutrients absorption and use efficiency, in addition to photosynthesis [54,55]. The significant increase of K present in plants cultivated at the two PH doses could also be responsible for the increase of root dry weight and fresh yield. In fact, increased K uptake, transport, and accumulation have been related to cell expansion, membrane trafficking, auxin homeostasis and cell signaling, and, above all, root growth [56]. Both the PH doses also significantly decreased the leaf brightness (L*) CIELab parameter, while enhancing net CO_2_ assimilation rate (ACO_2_). These results could depend on the increase, although it was not significant, of polyphenols which caused a more intense color of lettuce, as previously seen by Carillo et al. [57], ameliorating the antioxidant defense system of the plants and thus the protection of photosynthetic machinery and efficiency (Figure 5).

The PH, exerting an equal effect on the aerial part independently of dose, at 2.5 mL L^−1^ dose, was more effective in increasing the root to shoot ratio than the higher, therefore specifically increasing root weight and/or dimension. This was likely related to the higher ability of plants grown at lower PH dose to uptake nitrate and calcium. It is likely that PH at a lower dose could re-shape the root system architecture (RSA), even improving the root foraging ability of plants and thus increasing the NO_3_^–^ absorption and use efficiency [58]. Moreover, the increased root uptake ability also increased the content of calcium, that, at higher concentrations in the nucleocytoplasmic compartment of root cells, might play a role in further signaling the improvement of primary root development and inducing meristem development and auxin homeostasis [59].

Instead, PH, at a 5.0 mL L^−1^ dose, was able to induce a significant increase of ACO_2_ even at 15 DAT compared to the other treatments. This made available to plants a higher amount of photosynthates that could be used for increasing the number of leaves and SPAD index, the latter significantly correlated with chlorophyll concentration, according to absorbance/transmittance measurements [60]. Moreover, the higher number of leaves and the significant increase in stomatal frequency on adaxial lamina, even in the presence of a non-significant increase of transpiration rates, proved that PH at a higher dose was able to increase not only photosynthetic efficiency but nutrient uptake and transport to aerial parts [61], as demonstrated by the significant increase of P and S, in addition to K in lettuce leaves.

## 4. Materials and Methods

### 4.1. Plant Material, Experimental Design, and Harvesting

The experimental trial was carried out in a passive ventilation greenhouse at the “Torre Lama” farm of Federico II University of Naples (Bellizzi, Italy; 40°37′14″88 N, 14°56′51″72 E, 62 m above sea level) in the spring 2016. On May 4, 2016, butterhead Trocadero lettuce (*Lactuca sativa* L.) plants (Pagano Costantino & F.lli, Scafati, Italy) were transplanted in pots (Ø = 16 cm, 3 L) in a growing medium containing 2/3 peat (Vigorplant, Fombio, Italy) and 1/3 perlite (Perlite Italiana, Corsico, Italy) (*v*/*v*). The plants were arranged at a distance of 25 cm between and within rows, with a planting density of 16 pt. m^−2^ and irrigated with a Hoagland nutrient solution balanced in macro and micronutrients as follows: 8.2 mM NO_3_^−^, 0.7 mM P, 1 mM S, 2.7 mM K, 2.9 mM Ca, 0.7 mM Mg, 1 mM NH_4_^+^, 20 μM Fe, 20 μM B, 9 μM Mn, 1.6 μM Zn, 0.3 μM Mo, and 0.3 μM Cu (1.4 dS m^−1^). The nutrient solution was supplied through a drip irrigation system with 2 L h^−1^ drippers. A randomized block design was adopted. The plants were treated with a legume-derived PH biostimulant (Trainer^®^, Hello Nature, Rivoli Veronese, Italy) whose composition is detailed in the work of Rouphael et al. [46]. The PH was sprayed weekly from the seventh day after transplant (DAT) for five weeks with a solution containing 0.0 (Control), 2.5 mL L^−1^, or 5.0 mL L^−1^ of Trainer^®^ using a 10 L stainless steel sprayer. The dose of 2.5 mL L^−1^ was based on the manufacturers’ recommendations, while the double dose (5.0 mL L^−1^) was chosen assuming additive biostimulant action since leafy greens have a short crop cycle and it would be economically feasible. Each experimental treatment included nine plants and was replicated three times (*n* = 3).

At the end of the experiment (June 9, 37 DAT), all plants were sampled and separated into leaves, stems, and roots to determine biometric parameters and quality parameters. The leaves and stems were weighed for each plant to determine fresh biomass (g plant^−1^). A subsample of leaf tissue was frozen in liquid nitrogen and stored at −80 °C for qualitative analysis. All tissues collected, including roots, were dried in a forced ventilation oven at 70 °C until a constant weight was reached to determine dry biomass (g plant^−1^). The dried leaves were ground with an MF10.1 cutting head mill (IKA^®^, Staufen im Breisgau, Germany) and sieved to 0.5 mm. Then, the derived biometric indices, such as the shoot-to-root ratio and the percentage of dry matter (dry biomass/fresh biomass × 100), were determined. The leaf area per plant was measured using an electronic area meter (Li-Cor3000, Li-Cor, Lincoln, NE, USA).

### 4.2. Leaf Gas Exchange, SPAD Index, and Leaf Color

Leaf gas exchange was determined using a portable LCA-4 analyzer (ADC BioScientific Ltd., Hoddesdon, UK) set to ambient values of PPFD (700 µmol m^−2^ s^−1^), relative humidity (55%), and CO_2_ (365 ppm) with an airflow rate of 400 mL s^−1^. Measurements were taken at 15, 23, and 37 DAT, between 11:00 a.m. and 2:00 p.m., on healthy, fully expanded leaves of 3 plants per replicate per treatment. The net CO_2_ assimilation rate (ACO_2_; μmol CO_2_ m^−2^ s^−1^), transpiration (E; mol H_2_O m^−2^ s^−1^) and water use efficiency (WUEi = ACO_2_/E; μmol CO_2_ mol^−1^ H_2_O) were determined.

At 15 DAT, on the same leaves used for the determination of the leaf gas exchange, the measurements of the SPAD index were made using a portable SPAD-502 analyzer (Konica Minolta Co., Ltd., Osaka, Japan).

At 37 DAT, the colorimetric coordinates L*, a*, and b* of the leaves were determined as described by El-Nakhel et al. [62] using a Minolta Chroma meter CM-2600d (Minolta Co., Ltd., Osaka, Japan), calibrated with the corresponding Minolta standard.

### 4.3. Total Nitrogen and Macroelements

The total nitrogen concentration in the leaves was determined according to the Kjeldahl method described in detail by Formisano et al. [63]. Briefly, one gram of ground leaves was mixed with 7 mL of sulfuric acid (96%), a catalyst, and 10 mL of 30% hydrogen peroxide, and digested to complete mineralization (30 min at 420 °C). Sodium hydroxide of 33% was added to the mineralized samples to remove excess sulfuric acid and then steam distilled with boric acid. Quantitative determination of the ammonia produced was carried out by acid-base titration with 0.1 N sulfuric acid with methyl red and bromocresol green. The titration volume was used to calculate the percentage of total nitrogen converted to mg nitrogen g^−1^ dry weight (dw).

Macronutrient and nitrate concentrations were determined by ion chromatography coupled to an electrical conductivity detector, as described by Ciriello et al. [64]. An aliquot of 0.25 g of dry ground sample was extracted in ultrapure water at 80 °C for 10 min and then centrifuged. The supernatant was filtered and processed on the ion chromatograph (ICS-3000, Thermo Scientific™ Dionex™, Sunnyvale, CA, USA) using a sample injection volume of 25 µL. The determination of anions (nitrate, P, and S) and cations (K, Ca, and Mg) was performed via isocratic and gradient separation, respectively. Columns and pre-columns for macroelement separation and the software used for mineral integration (Chromeleon™ 6.8 Chromatography Data System) were purchased from Thermo Scientific™ Dionex™ (Sunnyvale, CA, USA). Mineral concentrations were expressed as mg g^−1^ dw, save for nitrate that was expressed as mg kg^−1^ fw, based on each sample dry weight. The analyses for the determination of total nitrogen and minerals were performed in triplicate.

### 4.4. Hydrophilic Antioxidant Activity, ABTS, and Total Phenols

Hydrophilic antioxidant activities and ABTS were determined by two different extraction procedures according to the methods described by Ciriello et al. [65].

Hydrophilic antioxidant activity (HAA) was evaluated using the DMPD method, which is based on the use of chromogen 4-amino-N,N-dimethylaniline dihydrochloride. In an acidic environment and the presence of an oxidant, this compound forms a resonance-stabilized radical cation characterized by an intense red color with an absorbance peak at 505 nm. The measured hydrophilic antioxidant activity was expressed in mmol equivalents of ascorbic acid 100 g^−1^ dw by converting the values obtained based on a calibration curve.

The ABTS assay determines the cationic radical 2,2′-azino-bis(3-ethylbenzothiazolin-6-sulfonic acid). A solution is prepared by mixing 7 mL of ABTS and 2.46 mM of potassium persulfate and placed in the dark for 18 h to stabilize the ABTS^•+^ radical. The spectrophotometric reading is performed at 734 nm. The measured antioxidant activity of ABTS was expressed in mmol equivalents of Trolox (6-hydroxy 2,5,7,8-tetramethyl carboxylic acid) 100 g^−1^ dw.

The optical readings of the extracts were taken 10 min after sample preparation, using the Hach DR 4000 UV-Vis spectrophotometer (Hach Co., Loveland, CO, USA) at 505 and 734 nm for the DMPD and ABTS assays, respectively.

The quantification of the total phenol concentration of the leaves was determined according to the Folin–Ciocalteu method described by Ciriello et al. [66]. 250 mg of freeze-dried and finely ground leaves were mixed with 10 mL of 60% methanol, shaken (15 min at 4000 rpm), and centrifuged. The supernatant was taken and mixed with phosphotungstic acid and phosphomolybdic acid, and the absorbance was read at 765 nm with a UV-vis spectrophotometer (Hach DR 4000; Hach Co., Loveland, CO, USA). Gallic acid was used as the standard, and the phenolic concentration was expressed as mg equivalents of Gallic acid 100 g^−1^ dw.

### 4.5. Microscopy

At 37 DAT, twelve fully expanded leaves were sampled per each treatment, and the median portion was fixed in F.A.A. (38% formaldehyde, glacial acetic acid, 50% ethanol solution, 5/5/90 by volume) for several days. Leaf samples were then dehydrated (up to 95% ethanol) and embedded in JB4 resin (Polysciences, Hirschberg, Germany). Cross-sections (5 μm thick) were cut by means of a rotative microtome and stained with Toluidine blue [67]. Digital microphotographs under light microscopy (BX51, Olympus, Hamburg, Germany) were taken (EP50; Olympus) to quantify anatomical traits with the software Olympus CellSens 2.3. The following parameters were measured: thickness of lamina and mesophyll (in five regions); palisade and spongy parenchyma density (as percentage of tissue occupied by intercellular spaces over a given surface, in four regions of the lamina); stomata frequency on the adaxial and abaxial epidermis (as number of stomata per mm along three epidermal transects).

### 4.6. Statistics

Data are presented as mean ± standard error, and all statistical analyses were performed with SPSS 26.0 software (IBM, Armonk, NY, USA). Comparison of the mean effects of the factor considered (Biostimulant-B) was performed by one-way analysis of variance (ANOVA), while statistical significance was determined using the Tukey’s HSD test at the *p* < 0.05 level. The loading plot and score plot of all analyzed parameters was determined after principal component analysis (PCA) by using Minitab^®^ 18 statistical software (Minitab LLC, State College, PA, USA) [68]. The heat map results were calculated as Logarithm base 1.5 (Log_1.5_) of the treatment to control ratio and visualized using a false-color scale, with red indicating an increase and blue a decrease of values [69].

## 5. Conclusions

The use of biostimulants represents a sustainable strategy to respond to the challenge imposed on the agricultural sector to provide food to the growing population while reducing chemical inputs in crop systems. Under the experimental conditions of our study, the comparison of use of three different PH concentrations (0.0, 2.5 and, 5.0 mL L^−1^) also provided an opportunity to better evaluate the efficacy of this biostimulant at different doses. It is noteworthy that, notwithstanding, the PH was supplied as a foliar spray in addition to the common effects seen at the two different doses, while in the higher root dw in particular, the 5.0 mL L^−1^ dose was effective both on leaves on roots, inducing an enhancement of photosynthetic activity in the early phase of growth (15 DAT), thus supplying carbon skeletons useful for increasing the number of leaves and their efficiency (higher SPAD), but also boosting nutrient uptake and transport to leaves, as demonstrated by the increase of P and S, in addition to K, whereas the 2.5 mL L^−1^ dose exerted specific effects on roots, increasing their dimension and enabling them to better use the available nutrients, nitrate and Ca in particular, thus increasing NUE. Indeed, based on the results achieved, the application of PH, at both lower and higher dose, is a valid strategy to increase root biomass, leaf area, polyphenol content, and, therefore, the efficiency of nutrient use and plant growth. However, further experiments should be performed to evaluate the effects of the different doses of PH on products with shorter vegetative growth periods, such as baby leaves, to determine the correct dose of biostimulant able to boost their growth and performance.

## Figures and Tables

**Table 1:** The analysis of variance and mean comparisons for physiological parameters of greenhouse lettuce cultivated under three biostimulant doses.

Source of Variance	SPAD Index	ACO_2_			E			WUEi		
		(μmol CO_2_ m^−2^ s^−1^)			(mol H_2_O m^−2^ s^−1^)			(μmol CO_2_ mol^−1^ H_2_O)		
		15 DAT	23 DAT	37 DAT	15 DAT	23 DAT	37 DAT	15 DAT	23 DAT	37 DAT
Biostimulant										
0.0 (Control)	25.00 ± 0.13 b	7.25 ± 1.08 b	8.57 ± 0.06 b	7.51 ± 0.53	2.35 ± 0.28	3.25 ± 0.38	2.29 ± 0.17	3.07 ± 0.13	2.71 ± 0.3	3.29 ± 0.11
2.5 mL L^−1^	26.35 ± 0.61 ab	9.84 ± 0.16 ab	12.21 ± 0.21 a	8.27 ± 0.53	2.49 ± 0.27	3.64 ± 0.08	2.44 ± 0.25	4.05 ± 0.48	3.36 ± 0.05	3.47 ± 0.5
5.0 mL L^−1^	27.48 ± 0.55 a	12.31 ± 0.31 a	12.27 ± 0.83 a	9.37 ± 0.22	2.69 ± 0.35	4.18 ± 0.16	2.93 ± 0.08	4.79 ± 0.81	2.93 ± 0.11	3.2 ± 0.11
Significance	*	*	*	ns	ns	ns	ns	ns	ns	ns

ns and * indicate non-significant or significant difference at *p* < 0.05. Different letters within each column indicate statistically different groups according to the Tukey HSD post-hoc test (*p* = 0.05). Data are mean values ± standard error, *n* = 3. DAT: day after transplant, ACO_2_: net CO_2_ assimilation rate, E: transpiration, WUEi: water use efficiency.

**Table 2:** The analysis of variance and mean comparisons for leaf color, nitrate, antioxidant activities, and total phenols of greenhouse lettuce cultivated under three biostimulant doses.

Source of Variance	L*	a*	b*	Nitrate	HAA	ABTS	Total Phenols
				(mg kg^−1^ fw)	(mmol eq. Ascorbic Acid 100 g^−1^ dw)	(mmol eq. Trolox 100 g^−1^ dw)	(mg eq. Gallic Acid 100 g^−1^ dw)
Biostimulant							
0.0 (Control)	47.54 ± 0.23 a	–10.57 ± 0.50	27.66 ± 1.79	1450 ± 3.29 b	1.74 ± 0.03	4.07 ± 0.36	12.99 ± 1.87
2.5 mL L^−1^	44.89 ± 0.64 b	–10.15 ± 0.41	26.62 ± 1.19	1667 ± 39.40 a	1.72 ± 0.20	4.56 ± 0.08	14.70 ± 1.09
5.0 mL L^−1^	45.69 ± 0.01 b	–10.55 ± 0.35	27.31 ± 1.81	1540 ± 17.66 ab	1.43 ± 0.21	4.11 ± 0.15	13.80 ± 1.27
Significance	*	ns	ns	*	ns	ns	ns

ns and * indicate non-significant or significant difference at *p* < 0.05. Different letters within each column indicate statistically different groups according to the Tukey HSD post-hoc test (*p* = 0.05). Data are mean values ± standard error, *n* = 3. HAA: hydrophilic antioxidant activity, ABTS antioxidant activity.

**Table 3:** The analysis of variance and mean comparisons for leaf anatomical traits of greenhouse lettuce cultivated under three biostimulant doses.

Source of Variance	Intercellular Spaces in Palisade Parenchyma	Intercellular Spaces in Spongy Parenchyma	Total Lamina Thickness	Mesophyll Thickness	Stomatal Frequency on Adaxial Lamina	Stomata Frequency on Abaxial Lamina
	%		µm		*n* mm^−1^	
Biostimulant						
0.0 (Control)	44.50 ± 2.99	51.50 ± 3.99	324.10 ± 21.66	285.1 ± 19.89	1.36 ± 0.01 b	1.82 ± 0.28
2.5 mL L^−1^	42.50 ± 1.98	53.50 ± 1.99	284.50 ± 13.35	251.0 ± 13.53	1.53 ± 0.05 ab	1.30 ± 0.11
5.0 mL L^−1^	50.50 ± 2.00	59.40 ± 1.96	325.80 ± 9.73	287.0 ± 8.69	1.67 ± 0.10 a	1.26 ± 0.10
Significance	ns	ns	ns	ns	*	ns

ns and * indicate non-significant or significant difference at *p* < 0.05. Different letters within each column indicate statistically different groups according to the Tukey HSD post-hoc test (*p* = 0.05). Data are mean values ± standard error, *n* = 3.

## Data Availability

The data is contained within the article.
